# A Comparative Analysis of the Economic Burden of Breast, Colorectal and Lung Cancer-Related Premature Mortality in Slovenia: Value-Added Decomposition by Occupation-Gender Groups

**DOI:** 10.2478/sjph-2026-0019

**Published:** 2026-09-01

**Authors:** Tjaša Redek, Tanja Istenič, Petra Došenović Bonča, Ivica Ratoša, Andraž Perhavec, Daša Farčnik

**Affiliations:** University of Ljubljana, School of Economics and Business, Kardeljeva ploščad 17, 1000 Ljubljana, Slovenia; Institute of Oncology Ljubljana, Zaloška cesta 2, 1000 Ljubljana, Slovenia; University of Ljubljana, Faculty of Medicine, Vrazov trg 2, 1000 Ljubljana, Slovenia

**Keywords:** Cancer-related premature mortality, Indirect cost, Breast cancer, Lung cancer, Colorectal cancer, Occupation-gender value-added decomposition, Economic burden, prezgodnja umrljivost zaradi raka, posredni stroški, rak dojke, pljučni rak, rak debelega črevesa, razčlenitev dodane vrednosti po poklicu in spolu, ekonomsko breme

## Abstract

**Introduction:**

This paper aims to estimate the value-added loss attributable to premature deaths from breast, colorectal and lung cancer in Slovenia using a novel methodology using linked employer-employee data, enabling value-added decomposition by occupation-gender groups.

**Methods:**

The analysis is based on population-level microdata for the 2016–2022 period matched from several sources. Premature mortality costs are estimated using occupation- and gender-specific output losses rather than assuming uniform productivity across working-age individuals. We approximate intangible capital using each firm's occupational and gender structure and estimate productivity losses due to premature mortality by specific occupation, revealing differences in the economic footprint of different cancer types.

**Results:**

On average, the loss of value added from a premature death due to colorectal cancer is between €352,000 and €392,000, from lung cancer, on average, between €150,000 and €178,000 and from breast cancer, around €436,000. In total, given that Slovenia's GDP in 2024 was around €66 billion (€52 billion at constant 2015 prices), the total value-added loss associated with these three cancer types amounted to about 0.5% of Slovenia's 2024 GDP. Over the study period, premature mortality attributable to these cancers translated into an estimated cumulative (current and future) GDP loss of roughly €371 million.

**Conclusions:**

Results indicate that notable reductions in indirect costs could be achieved in Slovenia through efficient and effective measures to manage the burden of cancer, including cancer screening programmes, other primary prevention approaches, timely access to diagnostics and treatment, and return-to-work policies.

## Izvleček

**Uvod:**

Namen tega članka je oceniti izgubo dodane vrednosti, ki jo je mogoče pripisati prezgodnji smrti zaradi raka dojke, debelega črevesa in pljuč v Sloveniji z uporabo nove metodologije, ki temelji na uporabi povezanih podatkov o posameznikih in njihovih delodajalcih, kar omogoča razčlenitev dodane vrednosti po poklicnih in spolnih skupinah.

**Metode:**

Analiza temelji na mikro podatkih celotnega prebivalstva za obdobje 2016–2022, ki so bili povezani iz več virov. Stroški prezgodnje umrljivosti so ocenjeni z uporabo izgub proizvodnje, specifičnih za poklic in spol, namesto upoštevanja enake stopnje produktivnosti vseh delovno aktivnih posameznikov. Neotipljivi kapital je ocenjen na podlagi poklicne in spolne strukture vsakega podjetja, kar omogoča oceno izgub produktivnosti zaradi prezgodnje umrljivosti bolnikov z rakom glede na njihove specifične poklice, kar razkriva razlike v ekonomskem odtisu različnih vrst raka.

**Rezultati:**

Izguba dodane vrednosti zaradi prezgodnje smrti, povzročene z rakom debelega črevesa, v povprečju znaša med 352.000 in 392.000 evrov, zaradi raka pljuč v povprečju med 150.000 in 178.000 evrov, zaradi raka dojke pa okoli 436.000 evrov. Glede na to, da je bil BDP v Sloveniji leta 2024 približno 66 milijard evrov (52 milijard evrov po stalnih cenah iz leta 2015), je skupna izguba dodane vrednosti, povezana s temi tremi vrstami raka, znašala približno 0,5 % BDP Slovenije v letu 2024. V proučevanem obdobju je prezgodnja umrljivost zaradi obravnavanih treh vrst raka povzročila kumulativno (sedanjo in prihodnjo) izgubo BDP v višini približno 371 milijonov evrov.

**Zaključki:**

Rezultati kažejo, da bi lahko v Sloveniji z učinkovitimi in uspešnimi ukrepi za obvladovanje bremena raka, vključno s programi za zgodnje odkrivanje raka in drugimi pristopi primarne preventive, pravočasnim dostopom do diagnostike in zdravljenja ter podpornimi politikami za vrnitev na delo, znižali občutne posredne stroške proučevanih vrst raka.

## 1 INTRODUCTION

Cancer remains a major cause of premature mortality and lost productivity in Europe. In 2022, 1.15 million Europeans died of cancer, implying cancer accounted for 22% of all deaths. In Slovenia, the share of deaths due to cancer was almost 29% in 2022. Colorectal, lung and breast cancer contributed around 38% of all cancer-related deaths in the EU, and in Slovenia, a third. These cancers consequently accounted for a substantial share of deaths also among the working-age population (25–64 years). They accounted for 13% of all deaths and 41.6% of cancer deaths of the working-age population in the EU. In Slovenia, these three cancers accounted for 15.5% of all deaths and almost 39% of cancer deaths among the working-age population (1).

While any death is a personal tragedy, years of life lost also represent a significant part of the economic burden of cancer. The latter captures direct health and non-health costs of cancer, as well as indirect costs resulting from productivity losses that reflect the value of lost current and future paid and also unpaid production. Estimates for Europe show that productivity losses due to early death and lost working days represent 35–41% of the total economic burden (2, 3). Productivity losses are highly relevant for policymaking on prevention, screening, and timely treatment, because they connect health shocks to macroeconomic performance through GDP losses from reductions in labour supply and in human and physical capital accumulation (4).

Studies on the economic burden of cancer consistently identify premature mortality as a major driver of productivity losses (2, 3, 5). Estimates of cancer premature mortality show that lung, breast and colorectal cancer were the top three most costly cancers in Europe in 2020 (6). A study estimating paid and unpaid productivity losses due to premature mortality from cancer in Europe in 2018 also showed that the costliest cancer was lung, followed by breast cancer (7). In Ireland, projections of productivity losses in paid work and household activities due to cancer-related mortality until 2030 represent 1.4% of GDP annually, with lung, colorectal, and breast cancers accounting for the largest shares (8).

In Europe in 2020 (6), lung cancer-related premature mortality costs per cancer death amounted to €132,088 based on the human capital approach (hereinafter HCA) and €4,631 using the friction cost approach (hereinafter FCA). For breast and colorectal cancers, they amounted to €172,549 and €157,278, respectively, based on the HCA approach, with HCA/FCA ratios of 41.8 and 33.8, respectively.

Observed differences in estimates across studies can be linked to the time period studied and the methodology used. It is plausible to expect that newer studies show lower premature mortality costs, given that several studies have observed a notable decline in premature mortality over time (2, 9, 10). In terms of methodology, the most commonly used approaches to evaluate productivity losses resulting from disease, disability, and premature death are the above-mentioned HCA and FCA. It is important to note that the HCA takes the patient's perspective, treating any hour not worked as a lost hour, while the FCA takes the employer's perspective, assuming timely replacement of cancer patients who have either temporarily or permanently left the labour market (11). In addition to different approaches for identifying lost productive time due to illness, studies also employ diverse methods for assigning monetary values to productivity losses.

In a 2024 review of 281 studies addressing productivity losses in low- and middle-income countries (12), half of all studies evaluated productivity losses based on market wages, just under a third based their estimates on self-reported incomes, 15% on macroeconomic aggregates such as gross domestic product or gross national income per capita, and only a small proportion of studies based their estimates on willingness to pay for labour replacement, daily value of production, or other measures.

Most macro estimates of the productivity cost of cancer rely on national averages or sectoral means, leading to biased results (13) and obscuring heterogeneity across workers with different occupations and genders who generate value-added in production and, consequently, GDP in modern economies increasingly reliant on intangible assets.

This paper addresses this gap by evaluating and comparing the value-added loss attributable to premature deaths from breast, colorectal and lung cancer in Slovenia using a novel methodology and linked-employer employee data combined with population and death registry data. Building on a recent occupation-value-added decomposition framework (14), we estimate the costs of premature mortality using occupation- and gender-specific output losses, rather than assuming uniform productivity across working-age individuals. This approach enables us to capture how human capital composition and its gender structure drive enterprise-level productivity (14).

## 2 METHODS

This study relies on a micro-based method to quantify the GDP loss from premature mortality, explicitly accounting for how different human-capital structures, proxied by occupations, contribute to economic output. Unlike approaches that impute losses based on average sector productivity, we consider that firm productivity depends on its human-capital mix (i.e., intangible assets). In knowledge-driven economies, a large share of value-added stems from such intangibles; economies and firms with more intangible capital therefore forgo more value-added when mortality or morbidity decreases labour supply (15,16,17,18). We approximate intangible capital with each firm's occupational structure (17) and value losses by the specific occupations affected, yielding a more accurate estimate of output foregone due to premature mortality.

Because occupational groups by genders contribute unevenly to value added (16, 17), we decompose firm output by occupation-gender groups. Following the National Transfer Accounts (NTA) framework (19) and the EU Globalinto project (14), we apply a regression-based decomposition of firm real value-added without a constant, by regressing the firm's shares of workers on broad International Classification of Occupations (ISCO) groups split by gender. The estimated coefficients enable estimation of weights that attribute a firm's value-added to each occupation-gender group; dividing by headcount yields the group's value-added per employee. Lost value-added of deceased in different occupation-gender groups due to illness-related premature mortality, then measures the economic burden of studied illnesses, in our case, breast, colorectal and lung cancers.

The empirical analysis has three steps. First, using linked employer-employee administrative data, we map each firm's workforce into ten ISCO groups (20) and gender, resulting in 20 occupation–gender groups (10 × 2) (16, 18). Second, we regress firm real total value-added (*TVA_real,j_*) on these 20 workforce shares (using a regression with no constant – equation 1).

(1) $${\text{TVA}}_{\text{real,j}}=\sum_{\text{i}=1}^{20}\beta_{\text{i}}\cdot {\text{x}}_{\text{ij}}+\varepsilon_{\text{i}}$$

In equation (1), *i* ∈*{1,…,20}* denotes each occupation-gender group, *β_i_* represents the calculated regression coefficient specific to each group, *x_ij_* is the firm-specific share of employees within each group (, and *j* ∈*{1,…,n}* denotes individual firms included in the study. *TVA_real,j_* is calculated by subtracting the firm's cost of materials, goods and services and other operating expenses from gross profit from operations.

The estimated coefficients from the regression (reported in the Zenodo research repository) are used to estimate firm-level weights (*w_ij_*) for each group (equation 2).

(2) $${\text{w}}_{\text{ij}}=\frac{\beta_{\text{i}}\cdot {\text{x}}_{\text{ij}}}{\sum_{\text{i}=1}^{20}\beta_{\text{i}}\cdot {\text{x}}_{\text{ij}}}$$

The value added per employee for each of the gender-occupation groups (*VAL_real,i_*) is estimated using equation 3, where *TVA_real,j_* represents the total real value added of the firm, *w_ij_* indicates the share of the firm's predicted value added attributable to group *i*, and *L_i_* is the number of all employees from the *i-th* gender-occupation group.

(3) $${\text{VAL}}_{\text{real,i}}=\frac{\sum_{\text{j}=1}^{\text{n}}{\text{TVA}}_{\text{real,j}}\cdot {\text{w}}_{\text{ij}}}{{\text{L}}_{\text{i}}}$$

Third, we estimate lost productivity due to premature deaths. For each occupation-gender group, we take the number of breast, lung and colorectal cancer-related deaths, compute productive years lost before retirement, and multiply them by the group's value added per employee. Summing across deaths yields the total value-added loss.

The analysis is based on population-level microdata for the 2016–2022 period (21) matched from several sources using unique person identifiers. First, microdata on employees' age, gender, occupation and employer is obtained from the Statistical Registry of the Active Population. The population size ranges from 881,000–998,000 individuals annually. Occupations are defined based on ISCO level 1 codes. Second, data on date and cause of death, using International Classification of Diseases (ICD) 4-digit codes, are obtained from the Mortality data (causes of death) registry. This enabled us to identify deaths from colorectal (C18-20), lung (C34) and breast (C50) cancer. Third, individual (employee) data were matched with financial statements of their employers, which include data on value-added. Data is limited to firms with at least five employees, resulting in 100,613 firms included in the analysis. Given that data were pooled for the 2016–2022 period, value-added estimates are expressed in 2015 prices. Due to statistical-protection rules and small cell sizes, some ISCO major groups are either excluded from reported results (ISCO group 0) or data imputations are used (ISCO groups 1 and 8), given that the decomposition does not provide reliable estimates due to low numbers of observations, as reported in the results section. All datasets were matched by the Statistical Office of the Republic of Slovenia, and pseudonymised data were provided for this analysis.

## 3 RESULTS

The analysis is based on investigating the impact of premature cancer deaths on the entire working population in the 2016–2022 period, which encompasses 6.6 million individuals. Slovenia lost approximately 0.1% of its working population each year. Cancer was contributing close to 40% of all deaths (21). In total, 297 working women died of breast cancer in the investigated period, 696 individuals died of lung cancer (37.6 % were women), and 341 working individuals died of colorectal cancer; a third were women. On average, women who died of breast cancer in the investigated period were 50 years old, implying an average loss of 8.9 productive years per woman (not considering occupational structure). The individuals who died of colorectal cancers were on average 53.8 years old for men and 50.6 years old for women, implying a loss of 6.1 years of productive life if compared to the actual average retirement age for men and a loss of 8.3 years of productive life for women (disregarding occupational differences). Lung cancer decedents among men were on average 56.2 years old and 55.3 years old among women, implying an average loss of roughly 3.6 years of productive life if compared to the actual average retirement age (Table 1).

**Table 1:** Average age at death due to different cancer types, average age at retirement for men and women, 2016–2022.

	**Average age at death**					**Average age at retirement**	
	**Colorectal cancer C18–20**		**Lung cancer C34**		**Breast cancer C50**		

	**M**	**F**	**M**	**F**	**F**	**M**	**F**
1 – Managers	57.9	p	56.6	p	51.9	62.1	60.8
2 – Professionals	55.4	48.9	59.1	57.4	50.1	63.2	60.8
3 – Technicians and associate professionals	54.1	52.5	55.4	55.3	51.9	60.4	59.0
4 – Clerical support workers	51.7	52.5	57.3	54.6	49.4	59.3	58.5
5 – Service and sales workers	51.5	47.9	57.3	54.2	47.6	59.2	58.1
6 – Skilled agricultural, forestry and fishery workers	58.0	p	60.7	p	p	61.1	59.9
7 – Craft and related trades workers	52.9	p	55.0	p	50.8	59.2	57.5
8 – Plant and machine operators, and assemblers	53.0	p	55.0	p	p	58.8	57.2
9 – Elementary occupations	52.2	49.3	54.3	54.4	48.6	58.9	58.0
Total	53.8	50.6	56.2	55.3	50.0	59.9	58.9

Data: Own calculation based on data of the Statistical Office of the Republic of Slovenia (21).

Notes: Data for averages for selected occupations not provided due to statistical guidelines on data protection (cells marked p – protected). Data for male deaths due to breast cancer was not reported due to statistical data protection rules (number is less than 10 in total). The average age at retirement was calculated based on changes in activity status from active to retired recorded on December 31. Age at retirement is approximated by the age in the last year when the person was still active.

**Figure 1:**
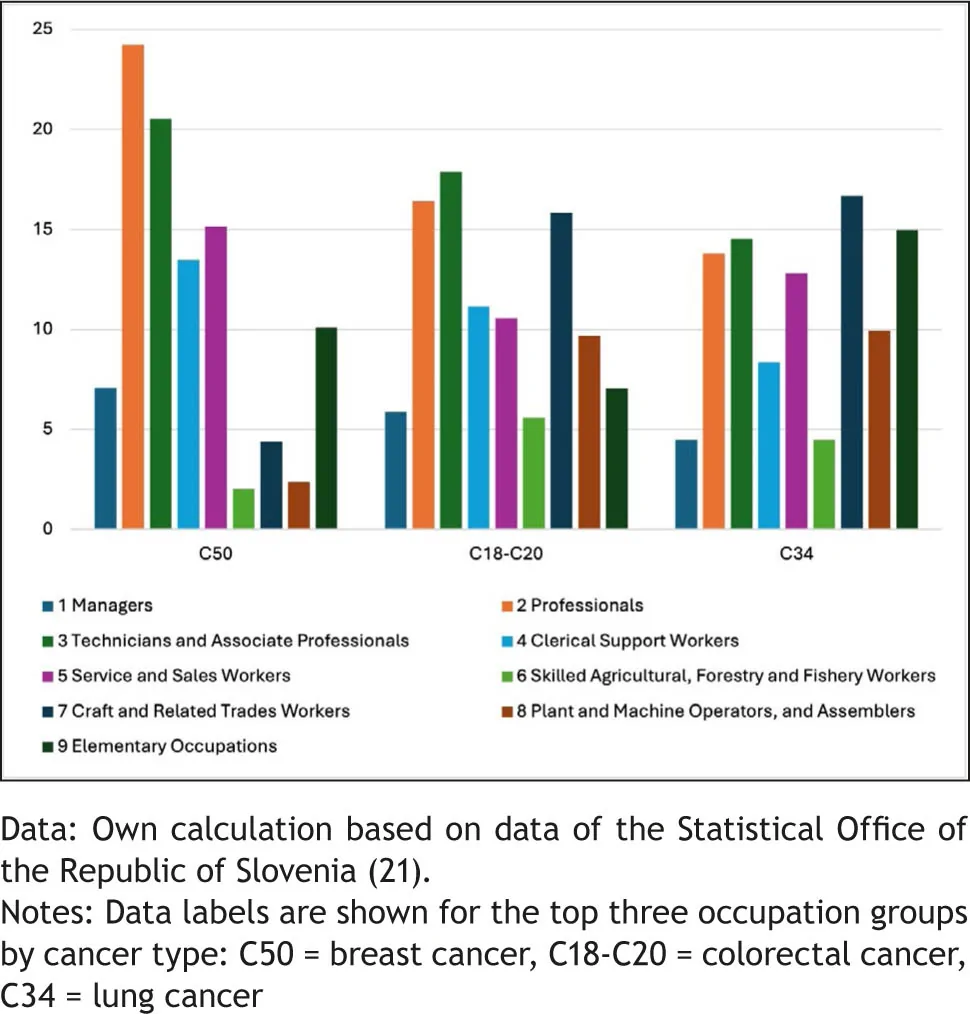
Occupational structure of deceased due to different cancer types (% of total deaths) at ISCO 1 level, 2016–2022.

Results reported in Table 1 disregard occupational differences. To correctly estimate the lost GDP, both the occupational and gender structures of decedents and the value-added contributions of each occupation need to be considered. Figure 1 shows the occupational structure of the deceased by cancer types. Deceased due to breast cancer were mainly active in the occupational groups of professionals, technicians and associate professionals. Deceased due to colorectal cancer were more evenly distributed, and were mainly active in the groups of technicians and associate professionals, craft and related worker occupations and professionals, while deceased due to lung cancer were most often from craft and related worker occupations, elementary occupations, as well as technicians and associate professionals, and professionals.

Table 2 presents the results of the estimation of productivity loss due to cancer-related premature mortality by cancer type. Total lost productive years are calculated as the lost productive years per person (column 1, which is the average retirement age minus the average age at death) multiplied by the number of deaths in the group. Total lost value-added (column 4) is estimated as the product of the value-added per person (column 3) and the total lost productive years (column 2).

**Table 2:** Estimation of value-added loss per occupational group, in total, in total per year and per person (values in 2015 prices).

	**Lost productive years per person (1)**		**Total lost productive years (2 = 1 × no. of deaths)**		**Value added per person in € (3)**		**Total lost value added in € (4 = 2 × 3)**	
**Colorectal cancer (C18–C20)**	**M**	**F**	**M**	**F**	**M**	**F**	M^***^	**F**

1 Managers^*^	4.2	10.8	50.5	86.2	86,620.8	77,316.5	4,370,128	6,666,053
2 Professionals	7.9	11.9	283.9	238.6	86,620.8	77,316.5	24,589,901	18,450,957
3 Technicians and Associate Professionals	6.3	6.4	251.3	135.2	76,952.1	29,753.4	19,335,190	4,022,284
4 Clerical Support Workers	7.6	6.1	122.2	133.5	126,022.6	21,765.9	15,402,764	2,905,453
5 Service and Sales Workers	7.7	10.2	131.0	193.2	7,408.7	37,131.5	970,558	7,173,593
6 Skilled Agricultural, Forestry and Fishery Workers	3.1	1.5	40.7	4.6	10,724.0	13,634.7	436,985	62,051
7 Craft and Related Trades Workers	6.3	7.5	322.7	22.4	20,165.9	38,672.4	6,506,987	866,444
8 Plant and Machine Operators, and Assemblers^**^	5.8	5.9	156.5	35.2	36,487.8	45,737.1	5,711,251	1,608,711
9 Elementary Occupations	6.8	8.7	81.3	104.5	23,315.9	35,815.5	1,895,584	3,742,339
**Total value added lost in €**			1,440.1	953.3			**79,219,347**	**45,497,885**
**Per person per year in €**							**55,011**	**47,724**
**Per person in €**							**352,086**	**392,223**

**Lung cancer (C34)**	**M**	**F**	**M**	**F**	**M**	**F**	M^***^	**F**

1 Managers^*^	5.5	3.0	116.5	29.8	86,620.8	77,316.5	10,094,765	2,301,881
2 Professionals	4.1	3.4	193.6	166.8	86,620.8	77,316.5	16,769,184	12,894,297
3 Technicians and Associate Professionals	5.0	3.6	270.6	171.2	76,952.1	29,753.4	20,823,597	5,093,227
4 Clerical Support Workers	2.0	3.9	53.8	122.2	126,022.6	21,765.9	6,781,606	2,659,478
5 Service and Sales Workers	1.9	3.8	86.1	164.7	7,408.7	37,131.5	638,062	6,115,666
6 Skilled Agricultural, Forestry and Fishery Workers	0.4	2.5	11.5	12.3	10,724.0	13,634.7	123,293	167,047
7 Craft and Related Trades Workers	4.2	2.3	441.1	22.7	20,165.9	38,672.4	8,895,279	877,183
8 Plant and Machine Operators, and Assemblers^**^	3.8	3.5	214.7	45.5	36,487.8	45,737.1	7,834,608	2,082,933
9 Elementary Occupations	4.6	3.7	231.1	199.2	23,315.9	35,815.5	5,387,924	7,134,536
**Total value added lost in €**			1,619.1	934.3			**77,348,319**	**39,326,248**
**Per person per year in €**							**47,773**	**42,092**
**Per person in €**							**178,222**	**150,100**

**Breast cancer (C50)**	**M**	**F**	**M**	**F**	**M**	**F**	M^***^	**F**

1 Managers^*^		8.9		187.3		77,316.5		14,483,051
2 Professionals		10.7		773.8		77,316.5		59,829,532
3 Technicians and Associate Professionals		7.0		427.7		29,753.4		12,726,759
4 Clerical Support Workers		9.1		363.9		21,765.9		7,920,277
5 Service and Sales Workers		10.5		472.8		37,131.5		17,556,831
6 Skilled Agricultural, Forestry and Fishery Workers		4.9		29.1		13,634.7		396,795
7 Craft and Related Trades Workers		6.6		86.3		38,672.4		3,335,642
8 Plant and Machine Operators, and Assemblers^**^		9.8		68.4		45,737.1		3,126,975
9 Elementary Occupations		9.5		284.2		35,815.5		10,179,596
**Total value added lost in €**								**129,555,458**
**Per person per year in €**								**48,098**
**Per person in €**								**436,214**

Data: Own calculation based on data of the Statistical Office of the Republic of Slovenia (21).

Notes:

* Data for managers (ISCO group 1) was imputed by assuming the same value as for professionals (ISCO group 2).

** Data for plant and machine operators and assemblers (ISCO group 8) were imputed, and the average value for all occupations excluding managers was used.

*** For breast cancer, we report estimates for women only.

The results show that, on average, the loss of value-added from a death from colorectal cancer is between €352,000 and €392,000, from lung cancer, on average, between €150,000 and €178,000 and from breast cancer, due to significantly younger age at death compared to the other two cancers, the value-added loss is around €436,000.

**Figure 2:**
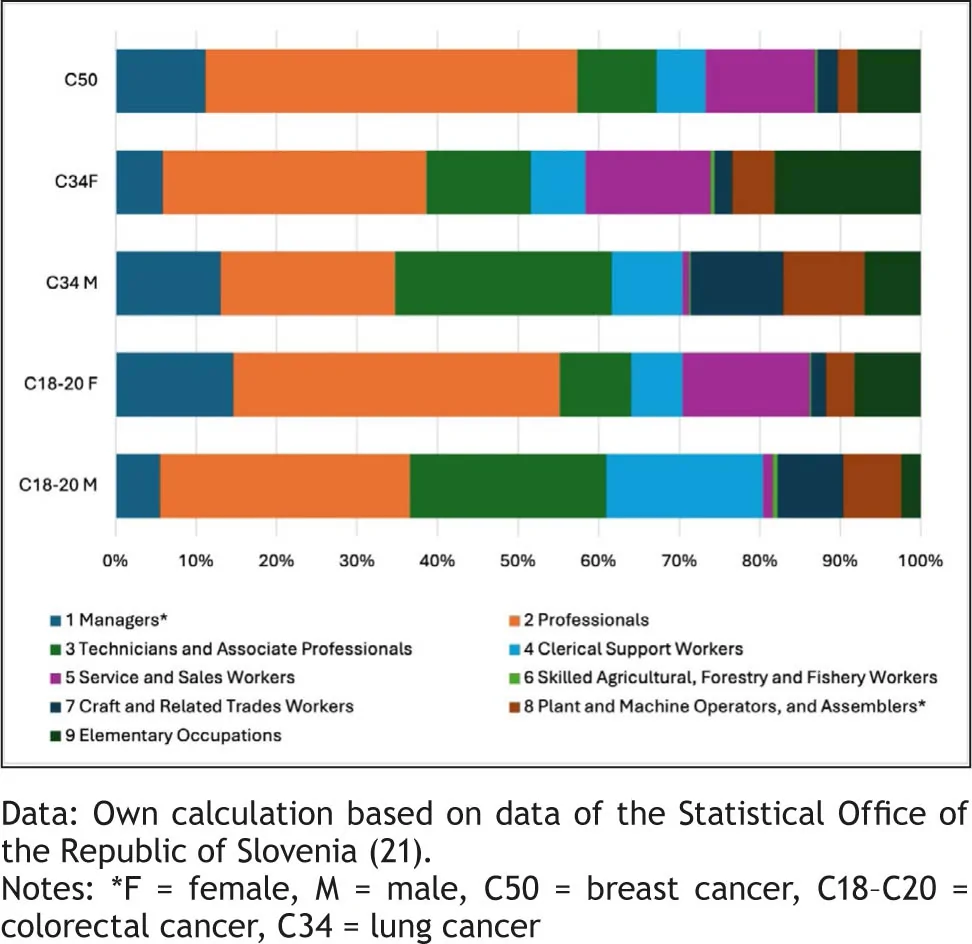
Decomposition of value added lost due to deaths and lost years by occupation.

Figure 2 also presents the contribution of each occupation-gender group to the total value-added loss by cancer type.

Among women for all cancer types, the biggest share of the loss comes from the loss of human capital in professionals, who create a high value-added and die relatively young, while retiring on average 10 years after the average age at death, implying a significant number of lost years. A similar conclusion can be drawn for men for colorectal cancer. The share of lost value-added due to the loss of human capital among technicians and associate professionals is high for men, namely around a quarter of total value-added lost for both lung and colorectal cancer. These high shares reflect primarily the high representation of this occupation among the employed who died.

## 4 DISCUSSION

The value-added decomposition by occupation and gender is a refined method of estimating premature mortality-related productivity losses. If compared with existing research using traditional human-capital methods, an analysis across EU countries (13) found that premature deaths in working ages led to productivity losses averaging 1.18% of GDP (2015), ranging from about 0.68% to 3.2% in some Eastern European countries. For Slovenia, production losses attributable to premature mortality in PPP in 2015 were estimated at €444 million for all premature deaths. In another study (6), cancer-related premature mortality costs amounted to €156 million in Slovenia in 2020, with breast, colorectal and lung cancer-related premature mortality costs amounting to €173, €157 and €132 thousand per cancer death, respectively. In another study (22), loss due to breast cancer per death among women was estimated at around €170 thousand, and lung cancer among men per death at €220 thousand. We also observe differences by cancer types in our analysis, and estimated premature mortality costs range from €150 to 436 thousand (not PPP) euros per death. A broader range can be explained by our use of occupation- and gender-specific value-added rather than average per-worker output to estimate the value of lost productive years. We have, for example, shown that in the case of breast cancer in Slovenia, the loss of human capital can be attributed primarily to high-value-added creators who have died relatively young but have worked primarily in the occupation group of professionals that have an above-average length of productive life.

This paper makes several contributions to the literature. First, we provide the first Slovenia-specific comparison of value-added losses due to premature mortality from breast, colorectal, and lung cancers, using a unified decomposition based on micro-level data linked across multiple sources (23). Second, by attributing firm productivity to occupation-gender groups, we reveal the specific economic footprints of different cancer types. Namely, in the studied period, breast cancer deaths were concentrated among mid-career women in skilled occupations versus the older and more male-skewed profiles typical of lung or colorectal cancer. Third, results show possible notable reductions in indirect costs that could be generated in Slovenia through efficient and effective measures to manage the burden of cancer, including cancer screening programmes and other primary prevention approaches, timely access to diagnostics and treatment, and supportive return-to-work policies.

Despite presenting a novel approach to providing a more realistic estimate of premature mortality costs, this value-added decomposition by occupation and gender is based on some assumptions. We proxy individual productivity with the average value-added of their occupation-gender group. We cannot control for deeper firm-level factors, such as synergies between worker types and team effects, or potential shocks from the loss of core human capital. This approach also treats losses as the full stream of future output and assumes that the lost human capital cannot be quickly replaced. While this can lead to overestimation of losses when there is excess labour supply, it better reflects the wider societal effects of premature mortality than approaches based on the FCA or the employer perspective. We also use a fixed retirement benchmark and rely on averages to compute productive years lost, while in reality some managers, for example, have been active beyond 80 years of age. Heterogeneity in actual exit ages introduces an additional source of possible bias in the estimates. Due to the small sample size and legislation regarding individual data protection, data for some ISCO groups were either excluded from the analysis or reasonable imputations were used. We also did not apply discounting or productivity growth to future years. This implies that we assumed that the future productivity growth rate and the economic discount rate are comparable. Annual snapshots used in these estimates can also differ from the real future workforce composition had the individual not died, suggesting another possible source of underestimation.

Several other limitations need to be noted. First, only the HCA was applied, as the FCA could not be estimated due to the lack of firm-level data on vacancies and replacement durations in Slovenia. The two approaches differ conceptually: the HCA estimates the full stream of forgone lifetime earnings from a societal perspective, whereas the FCA restricts productivity losses to the friction period required to replace a worker, reflecting the employer perspective. Reported HCA-to-FCA ratios of approximately 30–40 for similar cancer populations (6) suggest that our estimates likely overstate short-term employer productivity costs, while providing a more comprehensive measure of long-term societal productivity losses. Second, value added captures only paid market production. Consequently, losses in unpaid household production, informal caregiving and volunteer work are excluded, leading to an underestimation of the reported economic burden. Third, we identify lung cancer using ICD-10 code C34 rather than the conventional C33–C34 definition. Because cancers of the trachea (C33) account for only a small share of cases, the resulting underestimation is expected to be modest. Fourth, group-specific weights are estimated using a pooled OLS regression based on firm-level data for 2016–2022, anchored to the 2015 GDP price structure. This approach assumes that occupation-gender-specific contributions to value added remain stable over the study period. Future refinements could include panel specifications with fixed or random effects, as well as sensitivity analyses using alternative GDP real-growth scenarios, once longer and more complete micro-panel data become available. Finally, Slovenia's comparatively low wage dispersion, together with high female labour-force participation, is associated with less pronounced gender and inter-occupational differences in value added per worker. This should be considered when comparing the estimated magnitudes with those for EU labour markets characterised by greater wage dispersion.

A final interpretive caveat is warranted given the policy relevance of these estimates. Productivity-based valuations should not be interpreted as indicating which cancers are “less costly” or which deaths are less important to prevent. The main purpose of the estimates is to provide a detailed estimate in order to stress the importance of prevention. We also want to stress that because cancers disproportionately affect lower-paid occupations, retired persons, or groups with weaker labour-market attachment and lower estimated value-added losses, reliance on these metrics alone may amplify existing socioeconomic inequalities in cancer outcomes. In our view, productivity-based estimates should not be treated as stand-alone evidence, but rather as complementary information alongside epidemiological evidence, equity considerations, and direct measures of health-related quality of life, to further strengthen the case for equally accessible preventive action, screening, and treatment. In other words, while productivity loss can be assessed using detailed estimates, including value-added differences, these should not be used as a basis for assessing the economic justification of new health technologies, as this could lead to differences in access to these technologies. The identified limitations of this study point to several directions for future research. Relevant avenues would be to consider typical career paths of individuals and adjust estimates for the assumed (typical) career paths, to explore dynamic replacement times by occupation, and to use the approach to benchmark across countries, other cancer types, and cancer versus other chronic illnesses with a notable economic burden.

## 5 CONCLUSIONS

In total, considering that Slovenia's GDP in 2024 was around 66 billion euros (52 billion at constant 2015 prices), the total loss of value-added due to these three cancer groups was around 0.5% of 2024 GDP. Due to premature deaths attributable to the studied three cancer types, Slovenia lost a cumulative (future and current) GDP of around 371 million euros in the observed period. The occupation- and gender-specific decomposition identifies where productivity losses are concentrated: among professionals in high-value-added activities affected by breast cancer in mid-career, and among skilled craft and elementary-occupation workers affected by lung cancer. This level of granularity has direct policy relevance, as it indicates where targeted screening, earlier-diagnosis pathways and return-to-work support could generate particularly large economic benefits. When used alongside epidemiological and health-related quality-of-life evidence, these estimates provide a stronger empirical basis for informed decision-making and prioritising targeted cancer-control interventions.
